# HPV mRNA Is More Specific than HPV DNA in Triage of Women with Minor Cervical Lesions

**DOI:** 10.1371/journal.pone.0112934

**Published:** 2014-11-18

**Authors:** Sveinung Wergeland Sørbye, Silje Fismen, Tore Jarl Gutteberg, Elin Synnøve Mortensen, Finn Egil Skjeldestad

**Affiliations:** 1 Department of Clinical Pathology, University Hospital of North Norway, Tromsø, Norway; 2 Department of Medical Biology, Faculty of Health Sciences, The Arctic University of Norway, Tromsø, Norway; 3 Department of Microbiology and Infection Control, University Hospital of North Norway, Tromsø, Norway; 4 Institute of Clinical Medicine, The Arctic University of Norway, Tromsø, Norway; Queen Mary Hospital, Hong Kong

## Abstract

**Background:**

In Norway, repeat cytology and HPV testing comprise delayed triage of women with minor cytological lesions. The objective of this study was to evaluate HPV DNA and HPV mRNA testing in triage of women with an ASC-US/LSIL diagnosis.

**Materials and Methods:**

We used repeat cytology, HPV DNA testing (Cobas 4800) and HPV mRNA testing (PreTect HPV-Proofer) to follow up 311 women aged 25–69 years with ASC-US/LSIL index cytology.

**Results:**

Of 311 women scheduled for secondary screening, 30 women (9.6%) had ASC-H/HSIL cytology at triage and 281 women (90.4%) had ASC-US/LSIL or normal cytology. The HPV DNA test was positive in 92 (32.7%) of 281 instances, and 37 (13.2%) were mRNA positive. Of the 132 women with repeated ASC-US/LSIL, we received biopsies from 97.0% (65/67) of the DNA-positive and 92.9% (26/28) of the mRNA-positive cases. The positive predictive values for CIN2+ were 21.5% (14/65) for DNA positive and 34.6% (9/26) for mRNA positive (ns). The odds ratio for being referred to colposcopy in DNA-positive cases were 2.8 times (95% CI: 1.8–4.6) higher that of mRNA-positive cases. Compared to the mRNA test, the DNA test detected four more cases of CIN2 and one case of CIN3.

**Conclusions:**

The higher positivity rate of the DNA test in triage leads to higher referral rate for colposcopy and biopsy, and subsequent additional follow-up of negative biopsies. By following mRNA-negative women who had ASC-US/LSIL at triage with cytology, the additional cases of CIN2+ gained in DNA screening can be discovered. Our study indicates that in triage of repeated ASC-US/LSIL, HPV mRNA testing is more specific and is more relevant in clinical use than an HPV DNA test.

## Introduction

The goal in primary screening for cervical cancer is to detect and treat high-grade cervical intraepithelial lesions before invasive cancer develops [1]. In Norway, a cervical cancer screening program was introduced in 1995, recommending all women between 25 and 69 years of age to have a cytological cell sample (Pap-smear) collected every third year [2]. The Norwegian cervical cancer program recommends secondary screening with repeat cytology and an HPV test 6–12 months after the index diagnosis of an atypical squamous cell of undetermined significance (ASC-US) or low-grade squamous intraepithelial lesions (LSIL). Women with high-grade squamous intraepithelial lesions (HSIL) or ASC-US/LSIL with a positive HPV outcome are referred to colposcopy/biopsy immediately after triage. Women with a normal smear and a positive HPV test are recommended a repeat HPV test within 12 months, whereas women with an ASC-US/LSIL/normal smear with a negative HPV test are returned to the screening program at a three-year interval.

In Norway, cytological high-grade lesions are detected in 1.0–1.2% in each screening round of the national cervical cancer screening program [3]. The major challenge in any cervical cancer screening program is the management of minor cervical lesions such as ASC-US and LSIL [4]. Women with minor cervical lesions comprise a 4-fold volume of tests/visits for the health care system in comparison to women with high-grade cervical lesions [3]. In cytology-based screening several strategies have been assessed for women with minor cervical lesions, in combination with testing for human papilloma virus (HPV). One strategy is direct referral to colposcopy and biopsy in women with ASC-US/LSIL [5]. In reflex testing, women with ASC-US/LSIL are examined with an HPV test in the index cytology specimen, and positive cases are referred directly to colposcopy [6], [7]. A third strategy is repeat cytology after 6–12 months, while a fourth strategy is repeat cytology/HPV testing 6–12 month after primary ASC-US/LSIL diagnosis (delayed HPV triage) [8]. It is also possible to triage women with minor cervical lesions by HPV 16/18 genotyping or via biomarkers such as HPV E6/E7 mRNA, p16/Ki67, methylation and ProEx C [9]–[16].

In the ASC-US/LSIL triage study (ALTS) women were randomized to repeat cytology, direct referral to colposcopy or HPV triage [6], [7], [17]. After two years of follow-ups, there were no differences in detection rates of CIN3+ between the study arms. The HPV triage arm referred about half as many women to colposcopy as those with direct referral in ASC-US cases. Women with repeat cytology required at least two follow-ups leading to more colposcopic examinations than in the HPV arm [6], [7], [17]. In the ALTS study the prevalence of oncogenic HPV was too high (85%) to permit effective triage of LSIL using HPV DNA testing. Thus immediate referral to colposcopy was advocated for these women [17].

As only a small proportion (8–12%) of women with ASC-US or LSIL harbor high-grade histologically confirmed disease (CIN2+) [6]–[8], [17], [18], a test with high specificity would be desirable in secondary screening to avoid too many referrals to colposcopy [19]. Although HPV infection is a necessary factor in carcinogenesis, the majority of HPV infections are transient even in women with CIN2 after an ASC-US/LSIL diagnosis [20]. In general, HPV DNA tests generate more positive results than the HPV E6/E7 mRNA test [21]. This is because DNA tests detect the presence of the virus and will therefore also detect harmless transient infections, which are handled by the immune system, along with lesion regression [10]. The real cause of cervical cancer is not the HPV virus infection per se, but continuous over-expression of the viral oncogenes E6 and E7 from oncogenic HPV types [22]. Consequently, testing for the presence of HPV DNA is associated with a relatively low risk for underlying high-grade histological-confirmed intraepithelial lesion, even in women with ASC-US/LSIL [4].

In this study we performed a direct comparison of an HPV mRNA and an HPV DNA test in secondary screening of ASC-US/LSIL related to referral rates for colposcopy, biopsy rates, histological outcomes and sojourn time back to the screening program.

## Results

For most women (81%) the index cytology represented the first ASC-US/LSIL diagnosis, whereas 10% and 9% of the women had a history of one or two to five ASC-US/LSIL diagnoses, respectively. The positivity rate of the HPV DNA test was 36.7% (114/311) relative 18.3% (57/311) for the HPV mRNA test (Table 1).

**Table 1:** Cytology at triage by HPV test positivity.

Cytology at triage	Number	HPV DNA pos	HPV mRNA pos
Normal	142	25 (17.6)	9 (6.3)
Inconclusive	7	0 (0.0)	0 (0.0)
ASC-US/LSIL	132	67 (50.8)	28 (21.2)
ASC-H/HSIL	30	22 (73.3)	20 (66.7)
**Total**	**311**	**114 (36.7)**	**57 (18.3)**

Thirty of the 311 women had ASC-H/HSIL cytology at triage, and 22, 20 and 8 of the ASC-H/HSIL cases were HPV DNA positive, HPV mRNA positive and HPV DNA/mRNA double negative, respectively. All 20 HPV mRNA positive cases were DNA positive. Among ASC-US/LSIL cases at triage, 68 were HPV positive for one or both tests: 27 were double positive (DNA+/mRNA+), 40 were DNA+/mRNA- and one woman was DNA-/mRNA+.

A total of 281 women (90.4%) had ASC-US, LSIL, normal or inconclusive cytology at triage. Of these 281 women, 92 (32.7%) were HPV DNA positive and 37 women (13.2%) were HPV mRNA positive (Table 2). Among these 281 women, 16 were positive for HPV type 16 (DNA and/or mRNA), 15 for HPV DNA and 14 for HPV mRNA. Similar estimates for nine HPV type 18 positive women (HPV 16 negative) were seven and six for HPV DNA and HPV mRNA, respectively. Most discordant pairs were seen for other HPV types (HPV 16 and 18 negative), where 70 out of 71 were HPV DNA positive, relative to 17 HPV mRNA-positive women (Table 2).

**Table 2:** Concordance between HPV mRNA and HPV DNA types.

Triage test	HPV DNA				
HPV mRNA	Negative	HPV 16*	HPV 18**	HPV Others***	Total
HPV mRNA negative	186	2	3	53	244
HPV mRNA 16*	0	13	0	1	14
HPV mRNA 18**	2	0	4	0	6
HPV mRNA Others***	1	0	0	16	17
**Total**	**189**	**15**	**7**	**70**	**281**

*HPV 16 and all other types.

**HPV 18, all other types except for HPV 16.

***All other types, except for HPV 16 and/or 18.

The status at triage by diagnostic test are displayed in Table 3. The direct referral rate to colposcopy/biopsy were 24% using HPV DNA test respective 10% using HPV mRNA test (p<0.001). Within 12 months after triage, 65 out of 67 HPV DNA positive had met for biopsy respective 26 out of 28 in the HPV mRNA group (Table 4).

**Table 3:** Status of triage by HPV-test.

Triage outcome	HPV DNA	HPV mRNA
Return to screening*	124 (44.1)	140 (49.8)
Follow-up by cytology**	90 (32.0)	113 (40.2)
Colposcopy/biopsy***	67 (23.8)	28 (10.0)
**Total**	**281 (100.0)**	**281 (100.0)**

*Normal/inconclusive cytology and HPV negative.

**Normal and HPV positive or ASC-US/LSIL and HPV negative.

***ASC-US/LSIL and HPV positive.

**Table 4:** Most severe histology of biopsies/cone specimen by screening test.

Histology	HPV DNA	HPV mRNA
Normal	19 (29.2)	5 (19.2)
CIN1	32 (49.2)	12 (46.2)
CIN2	10 (15.4)	6 (23.1)
CIN3	4 (6.2)	3 (11.5)
**Total** *	**65 (100.0)**	**26 (100.0)**

*Two of the HPV DNA positive and two of the HPV mRNA positive women with ASC-US/LSIL did not meet for colposcopy/biopsy.

The positive predictive values for CIN2+ were 21.5% (14/65) for DNA positive and 34.6% (9/26) for mRNA positive. The positive predictive values for CIN3+ were 6.2% (4/65) for DNA positive and 11.5% (3/26) for mRNA positive. The odds ratio (OR) for being referred to colposcopy were 2.8 (95% CI: 1.8–4.6) in the HPV DNA group compared with the HPV mRNA group. The increased referral rate resulted in an additional diagnosis of four more cases of CIN2 and one more case of CIN3.

Since we used the HPV DNA as a reference test, the sensitivity of the HPV DNA test is 100% (14/14) relative 64.3% (9/14, 95% CI: 39.2–89.4) for the HPV mRNA test. The corresponding estimates of specificity are 70.8% (189/267, 95% CI: 65.5–76.3) and 89.5% (239/267, 95% CI: 85.8–93.2).

## Discussion

Our study shows that HPV mRNA is more specific than HPV DNA in triage of women with repeated ASC-US/LSIL. A low positivity rate translates into a low referral rate for colposcopy, which is very appealing for triage situations. The referral rate for colposcopy was significantly higher for HPV DNA positive relative to HPV mRNA positive, winning only four more cases of CIN2 and one more case of CIN3. Thus, compared with the mRNA test, the use of DNA tests in triage more than doubled the workload for gynecologists and laboratories. As long as women with repeated ASC-US/LSIL and negative HPV mRNA tests are followed up with a new cytology after 12 months, very few cases of CIN2+ captured by DNA at triage will be lost [8], [18].

The higher specificity of the HPV mRNA test translates into 39 more direct referrals to colposcopy/biopsy after ASC-US/LSIL diagnosis and positive HPV DNA and negative HPV mRNA. Among these women, four cases of CIN2 and one case of CIN3 were diagnosed, in addition to 20 extra cases of CIN1 and 14 extra cases with normal histology (Table 4). All women with negative biopsies (normal/CIN1) need further follow-ups because of limited sensitivity of colposcopy/biopsy [23], [24]. The choice of test represents a trade-off between benefits (detected CIN2+), which are greater for HPV DNA test, and harms (unnecessary colposcopies/biopsies), which are smaller for HPV mRNA test.

The HPV DNA test has higher sensitivity for CIN2+ than the HPV mRNA test. Women with repeated ASC-US/LSIL and a negative HPV mRNA test cannot be considered free of CIN2+ and need follow-up with another smear within 12 months. In two recently published studies from Norway, this is also the case for women with a negative HPV DNA test in delayed triage of ASC-US/LSIL [8], [25]. As the data from the Norwegian cancer registry are complete from all cytology laboratories, the practice of following up HPV DNA negative cases of repeated ASC-US/LSIL is nationwide despite lack of national guideline of this practice [8], [25].

Several studies have compared performance of different HPV diagnostic tests in direct referral to colposcopy after an abnormal cytology of repeated borderline/mild dyskaryosis or worse [26], [27]. In the Predictors 2 study, colposcopy-negative women were considered free of intraepithelial cervical disease. In a subset of 670 women with mild or repeated borderline smears, Cobas 4800 had a sensitivity and specificity for CIN3+ of 100.0% and 23.0%, respectively, versus 80.9% and 67.9% for the mRNA PreTect HPV-Proofer test. If we apply these estimates to the Norwegian setting of delayed triage, an additional 296 colposcopies had to be performed using Cobas 4800 for winning 24 cases of CIN2 and 9 cases of CIN3, in comparison with the HPV mRNA test. In direct referral studies, the use of health resources is not considered an issue of unnecessary referrals nor an issue of overtreatment of CIN2 lesions that regress spontaneously [5], [20].

At the time of recruitment of Predictors 2 study, there were no guidelines for HPV testing. In 2010 the British recommendations implemented HPV testing in triage of women with borderline and mild dyskaryosis (reflex testing). Women with a negative HPV test are returned to screening. Women with a positive HPV test, but a normal colposcopy without having any biopsies collected, are also returned to screening. This is not the case in Norway. The Norwegian guidelines recommend follow-up of women with a negative cervical biopsy (www.kreftregisteret.no).

Within the British health care system all women with mild dyskaryosis or worse are referred to colposcopy. In Norway most of the cervical samples are collected by the general practitioner. The Norwegian health care system has limited resources allocated to colposcopy/biopsy. In Norway, the HPV test in delayed triage of women with ASC-US/LSIL index cytology is used to select women with a higher risk of CIN2+, thus reducing the number of referrals [8], [25], [28], [29].

Colposcopies are costly procedures and can cause psychological stress [30]. Histopathologic diagnoses of CIN1 and CIN2 in cervical biopsies are prone to poor inter-observer reproducibility [12], [31], and a high referral rate for colposcopy and a high biopsy rate will inevitably result in some degree of overtreatment [20], [32], [33]. Furthermore, conization increases the risk of premature birth and late abortions in subsequent pregnancies [34]–[36]. A reduced rate of referral to colposcopy will reduce the costs to the health care system, reduce overtreatment, reduce the negative impact of cervical treatment on pregnancy outcomes and reduce psychological stress for the women.

The strengths of this study are the direct comparison of clinical usefulness of two different HPV diagnostic tests applied in an unselected population within a national screening program. The study population is one with a low-risk of CIN2+, as none had HSIL or a diagnosis of CIN/ACIS or worse, and 19% of the women had a history of ASC-US/LSIL before index cytology.

We consider the small sample size (N = 311) and insufficient follow-up time in the cytology arm as limitations of our study. According to the Norwegian guidelines, women with normal cytology and a positive HPV result at triage should be followed up with a new cytology and HPV test within 12 months after triage. According to the Norwegian guidelines, women with repeated ASC-US/LSIL and a negative HPV result are returned to screening even though the risk of CIN2+ is more than 2.0% [8], [18], [25]. Our hospital recommends follow-up within 12 months of these women [18]. Because of limited follow-up time, we have incomplete numbers of biopsies in this subgroup.

The Norwegian follow-up strategy for ASC-US/LSIL reflects the natural history of duration of primary HPV 16/18 infections/lesion formation, as there is a 6–12 month window before triage is undertaken [37]. The most recent cytology was normal for all women before index ASC-US/LSIL. Therefore we consider the index cytology as a consequence of a primary HPV infection or a re-infection in women who have demonstrated clearance of HPV infections in the past. The interval from index ASC-US/LSIL to triage is a necessary time period for giving the immune systems an opportunity to clear the lesion [20]. This is especially important among younger women [38]–[41]. Study designs that practice reflex testing or immediate referral to colposcopy do not take the natural history of HPV infections/lesion formation into consideration and will diagnose and treat more lesions that otherwise will regress spontaneously [20], [42].

There is a trade-off in any screening program how to find the most efficient way of diagnosing the long standing CIN2+ which have the potential to progress to cervical cancer. HPV type 16 in particular, HPV 18 and HPV 45 have demonstrated the highest progression rates to CIN3 and cervical cancer over a 10–20 year perspective [43]–[47]. The concordance between the DNA test and the mRNA test diagnosing HPV 16 and HPV 18 infections was highly acceptable in this study. The discrepancy in HPV detection rates between the diagnostic tests were other HPV types, which have a much lower potential to progress to cervical cancer over the next decades. Therefore it is most important to diagnose CIN2/3 lesions specific to HPV 16, 18 and 45 as these lesions may progress faster to cancer [46], [47]. Even though less oncogenic high-risk HPV types are identified within cervical cancer, some data suggest the these HPV types have the potential to initiate the normal cell to progress to CIN2/3, and question the less oncogenic high-risk types' capacity to progress further from CIN into cervical cancer [48], [49].

Participation in a screening program is voluntarily. If there are too many “false” alerts, the program will lose legitimacy among women, which again may lead to lower attendance rates. In this respect we consider high specificity to be more important than high sensitivity in overall CIN2/3 detection. Within the referral algorithm for the Norwegian cervical cancer program, our study shows that the referral rate to colposcopy was more than doubled for the DNA versus the mRNA tested women, and the sojourn rate back to regular screening was significantly higher. The importance of a screening program is to treat the women in such a way that they remain confident in the program and continue attendance. So far these issues have not been discussed from the point of view of medicine, societal costs, ethics or women. There is a need for additional studies on head-to-head comparison of HPV tests in both primary and secondary screening that target different molecular sites.

## Materials and Methods

The Regional Committee for Medical and Health Research Ethics, Northern Norway, approved the study as a quality assurance study in laboratory work fulfilling the requirements for data protection procedures within the department (REK Nord 2012/276). Written consent from the patients for their information to be stored in the hospital database and used for research was not needed because the data were analyzed anonymously. The ethics committee specifically waived the need for consent.

Our study contains two different HPV tests to triage women with minor cervical lesions: the HPV mRNA test PreTect HPV-Proofer (NorChip AS), which detects E6/E7 mRNA (encoding the viral oncoproteins) of 5 HPV types, and the HPV DNA test Cobas 4800 (Roche), which detects 14 HPV types.

The HPV DNA test Cobas 4800 is designed as a qualitative single tube multiplex assay based on the real time PCR technology that simultaneously detects 14 high-risk genotypes. The assay identifies HPV type 16 and 18 with concurrent detection of twelve other HPV types (HPV-31, -33, -35, -39, -45, -51, -52, -56, -58, -59, -66, -68) using L1 primers, and ß-globin is used as an internal control. DNA is isolated from a scrape of cells from a woman's cervix and is subsequently mixed in reaction wells with primers and probes that specifically recognize and amplify HPV DNA. This reaction produces fluorescence, which is then measured to determine the presence of HPV in the cervical sample. Specialized pipetting technology combined with AmpErase enzymes reduces cross contamination risk (http://www.roche.com).

The HPV mRNA test PreTect HPV-Proofer is an E6/E7 mRNA-based real-time nucleic acid sequence-based amplification assay (NASBA), focusing on the reportedly most common oncogenic HPV types 16, 18, 31, 33 and 45 using specific E6/E7 primers for each HPV-type. To avoid false negatives due to degradation of mRNA, primers and probes against human U1A mRNA are included in the PreTect HPV-Proofer kit as a performance and integrity control. Artificial oligonucleotides corresponding to the viral mRNA were used as positive controls. Negative controls consisted of Rnase-free water and were included in each run (http://www.norchip.com/).

We extracted cervical cells from the LBC by the ThinPrep 2000 (Cytyc Corporation, Marlborough, MA, USA) for cytological examination. DNA/RNA was isolated from 5 ml of the leftover material and analyzed with PreTect HPV-Proofer. The mRNA testing was performed according to the manufacturer's instructions (NorChip AS). The HPV DNA testing (Roche Cobas 4800) was performed and in accordance with national guidelines for HPV testing [28]. We defined the HPV DNA test as the reference in the study.

Our department analyses annually 23 000 cervical cytology samples. Between January 1^st^, 1991, and March 31, 2013, 98 579 female residents were reported at the county level, with 494 400 valid cervical smears in the clinical database SymPathy. Within this database we identified 2394 women with at least one ASC-US/LSIL diagnosis from January 1^st^, 2010, through September 30th, 2012. After excluding women who had at least one HSIL diagnosis (n = 326) or cervical biopsy referral (n = 294) prior to index ASC-US/LSIL diagnosis, women who were 15–24 (n = 271) or 70–91 (n = 20) years of age at index ASC-US/LSIL, 1523 women were eligible for analysis. Furthermore, we excluded women with no follow-up (n = 199), who had direct biopsy (n = 79), who had triage less than 3 months (n = 65) or more than 18 months (n = 20) after index ASC-US/LSIL. After these exclusions 1169 women within the age span of the Norwegian cervical screening program and who had no prior HSIL or cervical biopsy constituted the preliminary study population.

Our study began on January 1^st^, 2012, at which time our department switched from HPV mRNA testing to HPV DNA testing in secondary screening of ASC-US/LSIL. Included were women who had their index ASC-US/LSIL cytology back in 2010 because of varying intervals in scheduled follow-ups. Our protocol expanded the time window and included all women who had a diagnosis ASC-US/LSIL over the last 3 to 18 months, different from the national screening program's recommended 6–12 month interval [8]. In the transition period to HPV DNA testing, our department only conducted cytology follow-ups in cases with sparse material in the cytology specimen or HPV mRNA testing when enough material was present. After excluding 376 women who were followed up with cytology only, and 482 women who had cytology and mRNA follow-ups only, our study population comprised 311 of 1169 eligible women who had cytology, mRNA and HPV DNA tests at triage. Outcome assessment was based on the histological result of biopsies, where CIN2+ was considered as the target disease and CIN1 and CIN0 (no CIN) were considered as absence of disease. Moreover, women with double negative liquid-based cytology (LBC) and HPV mRNA result were assumed to be free of disease. All women were followed through September 31, 2014 for end points.

All statistical analyses used SPSS version 21 to perform Chi-square tests, Mann-Whitney tests and survival analysis. A p-value <0.05 was considered statistically significant.

## Conclusions

Our study indicates that in triage of repeated ASC-US/LSIL, HPV mRNA testing is more specific and is more relevant in clinical use than an HPV DNA test.

## Supporting Information

Data S1 **Data file (SPSS): Data_PlosOne_ms-D-14-28018.**
(SAV)
